# CONTROL BELIEFS MEDIATE THE WITHIN-PERSON RELATIONSHIP BETWEEN EVERYDAY CONTEXT AND SUBJECTIVE AGE

**DOI:** 10.1093/geroni/igad104.2698

**Published:** 2023-12-21

**Authors:** Shenghao Zhang, Shevaun Neupert

**Affiliations:** Florida State University, Tallahassee, Florida, United States; North Carolina State University, Raleigh, North Carolina, United States

## Abstract

Appraisal and interpretation of personal experiences resulting from interaction with situational contexts might play an important role in shaping subjective age at within-person level, but it is unclear how this process unfolds. We propose that older adults evaluate situational contexts and reflect on their general psychological resources when determining their subjective age, and tested this proposal with volition of daily activities as proxy for appraisal of situational contexts and control beliefs as proxy for psychological resources. We hypothesize that appraising daily activities one engaged in as obligatory would deplete one’s perceived control and concomitantly make one feel older. Older adults (n=116) ranging in age from 60 to 90 (M=64.71) completed a nine-day daily diary study online, resulting in 743 total days. Participants reported their sociodemographic characteristics on Day 1 and major daily activities, volition of every reported activity, felt age, and control beliefs on Days 2-9. Results showed that on days when older adults felt that activities they engaged in were more of their own volition, they also felt more in control. Lower-level mediation result suggests that within-person control beliefs mediated the relationship between volition of daily activities and subjective age. Our findings suggest that older adults evaluate situational contexts and reflect on their control beliefs as a general psychological resource when determining their subjective age. These findings show the important role psychological resources play in determining subjective age from a within-person perspective and extended the within-person process proposed in previous theoretical models.

